# Pediatrics

**Published:** 1895-09

**Authors:** Maude J. Frye

**Affiliations:** Clinical instructor in diseases of children, Medical Department, University of Buffalo


### PEDIATRICS.

By MAUDE J. FRYE, M. D.,

Clinical instructor in diseases of children, Medical Department, University of Buffalo.

### SUMMER DIARRHEAS IN INFANTS.

ALLYN (*University Med. Magazine*, July, 1895,) concludes the summer diarrheas of infants are, primarily, instances of poisoning, the poisons being developed in the food, especially milk, or introduced into the stomach and bowels in some other way.

Apart from the decomposition of food, the most important factors contributing to their fatality are high temperature, a bad sanitary condition of the district, uncleanliness of the house and of its occupants, and immorality of the parents or guardians.

The occurrence of these cases should be foreseen in winter and early spring, and efforts made to secure better living quarters, and the highest degree possible of nutrition and health in the infants.

The most desirable thing is a trustworthy source of fresh milk supply. In the absence of the milk laboratories of Boston and New York, it is the personal duty of the physician to look carefully after the milk supplied to nursing infants. In young infants that have to be artificially fed I have had the best result with peptonised milk.

No infants should pass the summer in the city if they can be removed to the country under fairly good surroundings. This should be done as a precaution before July and before they are ill. If summer diarrhea develops, removal to a cooler place, where fresh milk can be had, should be accomplished as soon as possible.

---

### RATIONAL THERAPEUTICS OF CHOLERA INFANTUM.

Blech (*N. Y. Medical Journal*, March 2, 1895,) says prohibit all food, even the breast, for the first day. Wash out the stomach, unless the patient is too low, using hydrozone one-half ounce to sterilised water one pint. Repeat if necessary. Irrigate the bowels in every case, using hydrozone, ℥ii. to each quart of cold sterilised water. Repeat irrigations, if necessary, every two hours. Use morphine and strychnine, hypodermically, when indicated. Use alcohol spongings if irrigations fail to lower the temperature. Sweet, strong Russian tea is allowed the second twenty-four hours.

---

### QUININE IN PERTUSSIS.

Fischer (*N. Y. Medical Journal*, LXI., 19,) gives quinine three times a day, one centigram for the month, one decigram for the year, of each patient, using the muriate or sulphate. From twenty-seven cases he concludes it diminishes the number of attacks essentially in five days at the latest. It reduces even the most vehement whooping-cough to a mild bronchitis in twelve to fifteen days. It influences most favorably a possibly existing pneumonia. It often stimulates the appetite.

---

### ALCOHOL IN SICK CHILDREN.

Seibert (*Archives of Pediatrics*, XII., 5,) condemns its use in gastro-intestinal disorders, and even in typhoid, except in emergencies. Alcohol-fed pneumonic children convalesce more slowly than others treated without it, therefore he does not give it here except in threatened or actual collapse. In scarlatina he limits its use to an occasional dose of light wine or whisky in septic conditions induced by absorption from pharyngeal necrotic tissue. In malignant cases he has never seen the slightest good from its use. In diphtheria he uses it in large doses in heart weakness, though doubting its value. He believes that the amount given to sick children is, as a rule, in direct proportion to the physician's ignorance of the proper dietetic and hygienic management of the case.

---

### ADENOID GROWTHS IN CHILDREN.

Eustace Smith, (*Lancet*, May 25, 1895,) says naso-pharyngeal adenoid growths are common in infancy as well as in childhood. They may even be present at birth. At this early age it is uncommon for them to give rise to the ordinary symptoms of nasal obstruction. Such growths should always be suspected if the infant's nose be broad at the bridge and faintly dimpled on each side at the upper border of the inferior lateral cartilage, and especially if there be noticed any retraction in the inferior region of the thorax. Persistent snuffling in infants is no sign of syphilis in the absence of other symptoms, but rather of adenoids.

---

### TREATMENT OF CROUP BY CALOMEL SUBLIMATION.

Fruitnight (*Archives of Pediatrics*, XII., 6,) says this treatment is indicated when the symptoms show increasing laryngeal stenosis. The method of treatment is empirical and not deductive. In the average case the dose is fifteen grains, repeated hourly. The patient is kept in the vapor-saturated atmosphere for a period varying from ten minutes to half an hour, according to the gravity of the symptoms. The treatment is continued from one to twelve days, as indicated by the persistence of laryngeal obstruction. The evil consequences to be guarded against are salivation, diarrhea, and especially depression and prostration, accompanied by anemia. In over one hundred patients subjected to this method of treatment the writer has observed no case in which deleterious effects have attended or followed it. Calomel sublimation should be practised even though intubation has been done. In applying the remedy the chief principle is, that the vapor must not be dissipated throughout a large extent of atmosphere. The author mentions three pregnant women who persisted in inhaling the vapor of the calomel. In each abortion resulted.

